# Safety evaluation of the interchangeable use of robenacoxib in commercially-available tablets and solution for injection in cats

**DOI:** 10.1186/s12917-020-02553-7

**Published:** 2020-09-25

**Authors:** Mark C. Heit, L. Jay Stallons, Wolfgang Seewald, Caryn M Thompson, Céline E. Toutain, Stephen B. King, Rainer Helbig

**Affiliations:** 1 Elanco Animal Health, 2500 Innovation Way, Greenfield, IN 64140 USA; 2 Elanco Animal Health, Mattenstrasse 24a, CH-4058 Basel, Switzerland

**Keywords:** Robenacoxib, Tablet, Injection, NSAID, Cat, Safety, Interchangeable

## Abstract

**Background:**

Robenacoxib (Onsior™) is a non-steroidal anti-inflammatory drug developed for canine and feline use for the control of pain and inflammation. It is available as both tablets and solution for injection.

The objective of this study was to evaluate the safety of the interchangeable use of commercially available robenacoxib formulations when administered to cats orally using 6 mg tablets and subcutaneously using a solution for injection containing 20 mg/mL. Thirty-four naïve healthy 4-month old cats were enrolled in this 37-day study and were randomized to four groups (three robenacoxib and one control). One robenacoxib group received the maximum recommended dose (MRD) rate of each formulation, while the other two received two and three times this dose rate. The cats underwent three 10-day treatment cycles comprised of seven days of once daily oral administration followed by three days of subcutaneous administration. The third cycle was followed by an additional seven days of oral treatment. The control group received oral empty gelatin capsules or subcutaneous saline injections. Assessment of safety was based on general health observations, clinical observations, physical, ophthalmic, electrocardiographic and neurological examinations, clinical pathology evaluations, food consumption, body weight, and macroscopic and microscopic examinations. Blood samples were collected for toxicokinetic evaluation.

**Results:**

Blood concentrations of robenacoxib confirmed systemic exposure of all treated cats. All cats were in good health through study termination and there were no serious adverse events during the study. There were no changes in body weight, food consumption, ophthalmic, physical or neurological examinations during the study. Treatment-related abnormalities were of low occurrence at all doses and included injection site changes (transient edema with minimal or mild, subacute/chronic inflammation histologically) and prolongation of the QT interval. These findings were consistent with previously observed findings in studies with robenacoxib administered separately orally or subcutaneously in cats. Thus, there were no adverse effects that could be attributed specifically to the interchangeable use of oral and injectable robenacoxib.

**Conclusions:**

This 37-day laboratory study supports the safety of interchanging robenacoxib injection at a daily dose of 2 mg/kg with robenacoxib tablets at a daily dose of 1 mg/kg, or vice versa.

## Background

Effective management of pain and inflammation in veterinary medicine remains challenging, especially in cats. Potential reasons for this include difficulty in diagnosing and assessing pain in this species as well as the limited number of, and safety concerns with, analgesic drugs available for cats [1, 2]. Non-steroidal anti-inflammatory drugs **(**NSAIDs) produce analgesic and anti-inflammatory effects by limiting prostaglandin production through inhibition of the cyclooxygenase (COX) family of enzymes [3]. The coxib class of NSAIDs selectively inhibits COX-2 isoforms and several have been approved for use in veterinary medicine [4–9]. Robenacoxib is the only coxib licensed for feline use and has been demonstrated to be safe in both the tablet [5] and the injectable formulations [10, 11].

In the US, robenacoxib (Onsior, Elanco Animal Health) solution for injection and 6 mg tablets are approved for use in cats at a dose of 2 mg/kg subcutaneously and 1 mg/kg (0.45 mg/lb) orally, respectively, once a day, for a maximum of 3 days for the control of postoperative pain and inflammation associated with orthopedic surgery, ovariohysterectomy and castration. Robenacoxib tablets (1 mg/kg with a range 1–2.4 mg/kg) are licensed for oral use in cats in the EU for the reduction of moderate pain and inflammation associated with orthopedic surgery for up to three days, for the treatment of pain and inflammation associated with acute musculoskeletal disorders for up to six days and for long-term use for the treatment of pain and inflammation associated with chronic musculoskeletal disorders. Robenacoxib solution for injection (2 mg/kg) is licensed in the EU for subcutaneous use in cats for the treatment of pain and inflammation associated with orthopedic or soft tissue surgery in cats administered pre-surgery plus for up to 2 days after surgery.

Peri-operatively, administration of three consecutive days of the same formulation of robenacoxib for cats might not be the preferred or most practical option. For example, a pre-operative subcutaneous injection of robenacoxib followed by 2 days of oral tablets might be more convenient for a day surgery patient that needs continued treatment at home. Conversely, if the patient remains hospitalized for an extra day, the clinic might prefer to give 2 consecutive days of treatment by injection followed by one day of oral administration at home. Another example of when interchangeable use between formulations could be of benefit is for a cat in the EU receiving robenacoxib tablets once daily for the treatment of pain and inflammation associated with a musculoskeletal disorder that then develops a condition that requires surgery. In accordance with approved indications and directions for use, it might be preferable to use the solution for injection formulation peri-operatively before returning the cat to oral treatment for the ongoing management of the chronic musculoskeletal disorder.

This target animal safety study utilized a novel study design to establish the safety of the interchangeable use of robenacoxib tablets and robenacoxib solution for injection in cats. It was a component of the safety data package presented to the Food and Drug Administration’s Center for Veterinary Medicine (FDA-CVM) and the European Medicines Agency Committee for Medicinal Products for Veterinary Use (EMA CVMP) that demonstrated the ability to interchange between the different formulations of robenacoxib for cats in accordance with the approved indications and directions for use.

## Results

### Actual test article dosage

Mean test article dosages and dosage ranges achieved during the oral dosing periods are summarized in Table 1. The mean actual oral doses exceeded the daily target doses of 2.4, 4.8, and 7.2 mg/kg. In fact, due to the use of whole 6 mg tablets, the dose ranges achieved almost overlap (e.g., maximum 1X dose of 4.7 mg/kg, minimum 2X dose of 4.8 mg/kg). The SC doses were delivered at the exact dosage and individual dose volumes ranged from 0.2 to 0.3 mL, 0.4 to 0.7 mL and 0.5 to 1.1 mL for groups 2 (1X), 3 (2X) or 4 (3X), respectively.

**Table 1:** Mean (range) actual robenacoxib dosages during oral administration periods

**Group Number (Multiples of MRD)**	**Target Dose Level(mg/kg/day)**	**Male**		**Female**	
		**(mg/kg/day)**	**% of target**	**(mg/kg/day)**	**% of target**
1 (0X)	Placebo	0.00	NA	0.00	NA
2 (1X)	2.4	3.8 (2.4-4.7)	158	3.2 (2.4-4.7)	133
3 (2X)	4.8	6.0 (4.8-6.9)	125	5.9 (4.9-7.1)	124
4 (3X)	7.2	8.6 (7.2-9.6)	119	8.8 (7.2-10.0)	122

*MRD* maximum recommended dose, *NA* not applicable

Although gelatin capsules slightly delayed the initial dissolution of robenacoxib, by 40 minutes, approximately 93% had dissolved regardless of whether or not encapsulated (data not shown). It was therefore concluded that there was no impact of the gelatin capsule on rate and extent of robenacoxib absorption.

### Cageside and detailed clinical observations, body weight, food consumption, injection site monitoring

**Fig. 1:**
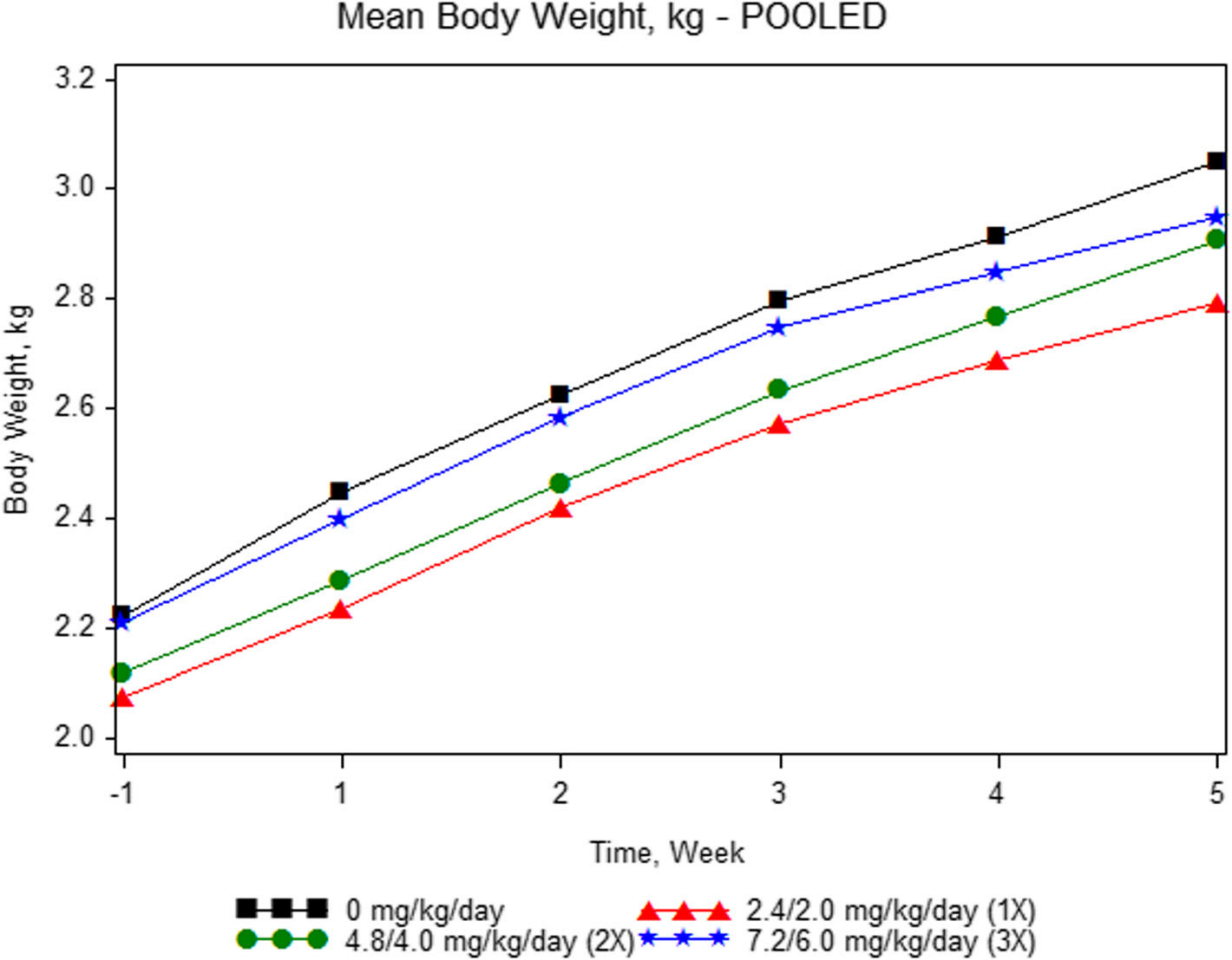
Body Weight vs. Time. Mean body weight and body weight gain did not differ between groups over the course of the 37-day study (*p*>0.10). The chart shows mean body weight for pooled sexes in each group

All animals were in good health until the scheduled necropsy and were included in all assessments. There were no significant treatment-related changes in body weight or food consumption (*p* > 0.10). Body weights for all groups are summarized in Fig. 1. Observations of soft feces were noted in 4/10 control animals, 3/8 1X animals, 5/8 2X animals, and 7/8 3X animals for up to four consecutive days. Emesis was observed once on Days 36 and 37 in a 2X male.

Edema was observed sporadically at the injection sites in control and robenacoxib-injected animals at 8 to 48 hours after dosing, except for a single injection site in one 2X and one 3X female where edema was observed up to 120 hours post injection. When pooled by gender, the occurrence of edema was significantly higher at 8 hours post dose for animals given 2X robenacoxib SC (*p* ≤ 0.10) and at 24 and 48 hours post dose for cats given 3X robenacoxib SC (*p* ≤ 0.01) than in control animals. There were no signs of injection site erythema, heat or pain in any control or robenacoxib-treated cat.

### Ophthalmoscopic examinations, physical and neurological examinations, electrocardiographic examinations

There were no treatment-related effects on opthalmoscopic, physical and neurological examinations. All animals appeared healthy during the physical examinations on Days 10 and 36 with the single exception of a 2X male that exhibited dry nasal discharge from both nares with clear lung auscultation on Day 10. Since this finding was resolved on Day 36 and was not observed in any other animals, it was considered unlikely related to treatment. No abnormalities were noted during neurologic examinations.

All animals were in normal sinus rhythm during all ECG examinations. All of the electrocardiograms were qualitatively normal exhibiting no arrhythmias, conduction disturbances or evidence of abnormal morphology. There was no statistically significant effect of the administration of robenacoxib on heart rate, although mean heart rate in the control group increased slightly from 289 bpm at baseline to 294 bpm post treatment, whereas in all treated groups, mean baseline heart rate was approximately 305 bpm and decreased to between 260 and 277 bpm (Table 2).

**Table 2:** Summary of means (± SD) of electrocardiographic examination variables

**Variable**	**Study phase**	**Control**	**1X**	**2X**	**3X**
Heart Rate [beats/minute]	baseline	288.8 ± 27.4	306.1 ± 20.4	304.0 ± 24.5	306.6 1± 7.2
	post-treatment	293.8 ± 42.0	259.8 ± 55.8	267.5 ± 24.8	277.4 ± 25.0
RR Interval [ms]	baseline	209.5 ± 20.8	196.8 ± 13.1	198.7 ± 17.3	196.4 ± 11.5
	post-treatment	209.0 ± 37.1	240.6 ± 51.5	226.1 ± 22.2	217.8 ± 19.6
QT Interval [ms]*	baseline	147.4 ± 12.1	139.4 ± 10.8	140.2 ± 12.1	138.9 ± 8.3
	post-treatment	143.1 ± 14.5	162.1 ± 25.7	160.2 ± 11.6	162.4 ± 13.7

*significant treatment effect (*p*=0.02)

A treatment-related increase in the QT interval was noted where average pretest values were approximately 140 ms in all groups which increased to approximately 160 ms (Table 2) in robenacoxib-treated groups, but not in the control group. When these data were pooled by sex and analyzed via ANCOVA using the terminal RR interval as a covariate, there was a dose-dependent increase in the QT interval at the two highest dose levels that was statistically significantly different from control group values (p = 0.0113, p = 0.0073, for the 2X and 3X groups, respectively). The percentage increases from controls for the QT interval based on least square mean values were 6.3% in the 2X group and 7.5% in the 3X group.

### Buccal mucosal bleeding time, hematology, coagulation, clinical chemistry, urinalysis

Pre-study BMBT ranged from 17 seconds to 1 minute and 21 seconds in all study animals. On Day 36, the times for all animals were within this range with the exception of one 2X male (3 minutes and 37 seconds). Because this was the only animal affected, it was considered unrelated to treatment. There were no significant differences in coagulation or urinalysis values between groups during the study (Table 3). There was a statistically significant treatment effect for phosphorus (*p* = 0.0675, Table 4) for which follow-up pairwise comparisons of pooled samples over all time points demonstrated a decrease in the 2X group relative to controls that was not considered clinically relevant. There were statistically significant interactions (i.e., treatment x sex, treatment x time) observed for various parameters (i.e., albumin/globulin ratio, aspartate aminotransferase, gamma-glutamyl transferase, triglyceride, potassium, eosinophils). None of these demonstrated consistent or dose-dependent changes and because of their sporadic nature, small magnitude and the fact that individual animal values remained within expected historical ranges, it was concluded that there were no treatment-related effects in hematology, coagulation, clinical chemistry, or urinalysis.

**Table 3:** Summary of post-treatment means (± SD) of hematology and coagulation variables (average of days 13 and 37)

**Variable**	**Reference range**	**Control**	**1X**	**2X**	**3X**
Leukocytes [10^3^/µL]	6.3 - 27.2	12.66 ± 3.76	13.95 ± 4.43	13.43 ± 3.10	14.76 ± 3.66
Erythrocytes [10^6^/µL]	5.36 - 10.78	7.61 ± 0.58	7.24 ± 0.88	7.58 ± 0.82	7.73 ± 0.88
Hemoglobin [g/dL]	7.5 - 14.4	10.08 ± 0.89	9.96 ± 0.98	9.99 ± 1.06	10.13 ± 1.15
Hematocrit [%]	22.5 - 41.6	30.74 ± 2.73	30.66 ± 3.36	30.17 ± 3.65	30.90 ± 3.81
MCV [fL]	34.3 - 47.5	40.45 ± 2.59	42.49 ± 2.78	39.86 ± 3.11	40.03 ± 2.65
MCH [pg]	11.9 - 15.1	13.28 ± 0.96	13.82 ± 0.81	13.21 ± 0.99	13.11 ± 0.65
MCHC [g/dL]	30.4 - 38	32.84 ± 1.85	32.53 ± 0.55	33.14 ± 0.65	32.80 ± 1.57
Platelets [10^3^/µL]	107 - 779	169.9 ± 134.8	253.7 ± 157.1	334.7 ± 155.9	301.4 ± 173.8
Neutrophils [10^3^/µL]	0 - 12.65	5.13 ± 1.79	5.28 ± 1.75	5.73 ± 1.34	5.46 ± 1.04
Lymphocytes [10^3^/µL]	2.18 - 13.48	5.73 ± 2.28	6.69 ± 2.61	6.16 ± 1.81	7.50 ± 2.64
Monocytes [10^3^/µL]	0 - 0.81	0.24 ± 0.19	0.27 ± 0.22	0.28 ± 0.14	0.26 ± 0.15
Eosinophils [10^3^/µL]^a^	0 - 1.31	0.96 ± 0.33	1.08 ± 0.40	0.94 ± 0.52	1.10 ± 0.40
Basophils [10^3^/µL]	0 - 0.03	0.004 ± 0.008	0.006 ± 0.011	0.010 ± 0.011	0.006 ± 0.007
Other Cells [10^3^/µL]	0 - 0.05	0.60 ± 0.90	0.62 ± 0.95	0.30 ± 0.71	0.44 ± 0.92
Absolute Aggregate Reticulocyte [10^3^/µL]	12.9 - 106.6	1.56 ± 4.14	4.02 ± 4.81	1.38 ± 4.14	1.45 ± 4.17
Absolute Punctate Reticulocyte [10^3^/µL]		2012 ± 602	2277 ± 658	1915 ± 702	2129 ± 555
APTT [sec]	11.2 - 18.7	17.12 ± 3.74	23.35 ± 16.35	16.68 ± 2.52	16.83 ± 2.52
Prothrombin Time [sec]	8.7 - 11	10.73 ± 0.41	11.03 ± 0.62	10.77 ± 0.56	10.84 ± 0.46
Fibrinogen [mg/dL]	152 - 278	175.4 ± 26.1	180.8 ± 37.0	180.3 ± 22.2	172.5 ± 16.3

*significant treatment effect  (*p*<0.1)

^a^significant treatment x sex effect (*p*<0.1)

**Table 4:** Summary of post-treatment means (± SD) of clinical chemistry variables (average of days 13 and 37)

**Variable**	**Reference range**	**Control**	**1X**	**2X**	**3X**
Sodium [mEq/L]	146 – 160	150.6 ± 1.1	150.3 ± 1.3	150.5 ± 1.4	150.6 ± 1.5
Potassium [mEq/L]^b^	4.6 - 7.4	5.55 ± 0.64	5.40 ± 0.24	5.33 ± 0.41	5.50 ± 0.43
Chloride [mEq/L]	112 – 123	116.00 ± 1.78	116.63 ± 1.50	115.88 ± 1.71	115.81 ± 1.97
Calcium [mg/dL]	9.6 - 11.5	10.25 ± 0.33	10.01 ± 0.31	10.16 ± 0.26	10.17 ± 0.26
Phosphorus [mg/dL]*	6.2 - 9.7	8.89 ± 0.88	8.86 ± 0.76	8.18 ± 0.83	8.66 ± 0.78
Alkaline Phosphatase [U/L]	50 – 186	102.9 ± 34.3	105.6 ± 40.6	94.5 ± 24.4	90.0 ± 19.5
Total Bilirubin [mg/dL]	0 - 0.3	0.19 ± 0.04	0.18 ± 0.04	0.19 ± 0.03	0.19 ± 0.03
GGT [U/L]^a,b^	0 – 2	1.10 ± 0.39	1.14 ± 0.43	1.33 ± 0.65	0.94 ± 0.05
AST [U/L]^a^	0 – 55	21.7 ± 5.5	22.2 ± 8.6	22.0 ± 4.1	22.8 ± 6.8
ALT [U/L]	27 – 103	59.4 ± 10.1	54.6 ± 9.2	56.6 ± 9.4	55.3 ± 10.5
Creatine Kinase [U/L]	0 – 1213	1007 ± 1250	1479 ± 1901	975 ± 903	1763 ± 2175
Urea Nitrogen [mg/dL]	15 – 35	26.1 ± 5.4	25.3 ± 2.7	24.8 ± 4.0	25.2 ± 3.9
Creatinine [mg/dL]	0.5 - 1.3	0.83 ± 0.14	0.86 ± 0.13	0.85 ± 0.12	0.79 ± 0.12
Total Protein [g/dL]	5.4 - 6.7	6.02 ± 0.32	5.93 ± 0.28	6.03 ± 0.35	6.10 ± 0.34
Albumin [g/dL]	2.9 - 3.7	3.46 ± 0.17	3.31 ± 0.21	3.46 ± 0.17	3.44 ± 0.18
Globulin [g/dL]	2.1 - 3.3	2.57 ± 0.28	2.62 ± 0.23	2.57 ± 0.28	2.66 ± 0.29
Albumin/Globulin Ratio^a,b^	0.9 - 1.5	1.36 ± 0.18	1.29 ± 0.14	1.36 ± 0.14	1.32 ± 0.16
Triglyceride [mg/dL]^a^	12 – 64	38.7 ± 19.8	40.1 ± 27.2	32.4 ± 9.1	37.7 ± 14.7
Cholesterol [mg/dL]	88 – 148	105.4 ± 19.0	105.0 ± 18.1	103.9 ± 14.2	112.6 ± 23.7
Glucose [mg/dL]	55 - 159	86.0 ± 10.3	85.1 ± 7.1	84.1 ± 9.0	83.1 ± 6.2

*significant (*p*<0.1)

^a^significant treatment x sex effect (*p*<0.1)

^b^significant treatment x time effect (*p*<0.1)

### Toxicokinetics of robenacoxib

For all dose groups, oral administration resulted in higher average 0.75 h concentrations and lower average 4 h concentrations than subcutaneous injection. Because data are not available following the first dose (Day 1), it is impossible to determine if any accumulation occurred between Day 1 and Day 4, however, based on comparing concentrations achieved 4 h post oral administration (Days 4, 7, 11, 14, 17, 31, 34, and 37) there was no accumulation observed after Day 4 (Table 5).

**Table 5:** Summary of plasma robenacoxib concentrations (mean ± SD) 0.75 and 4 h after repeated oral and subcutaneous (SC) administration

		**Group**					
		**1X**		**2X**		**3X**	
		**Hour(h)**		**Hour(h)**		**Hour(h)**	
		**0.75**	**4**	**0.75**	**4**	**0.75**	**4**
**Study Day**	**route**	**Mean ± SD(ng/mL)**	**Mean ± SD(ng/mL)**	**Mean ± SD(ng/mL)**	**Mean ± SD(ng/mL)**	**Mean ± SD(ng/mL)**	**Mean ± SD(ng/mL)**
4	oral	2593.1 ± 909.5	22.9 ± 11.6	5177.5 ± 2229.5	54.2 ± 28.5	5511.8 ± 3389.8	98.0 ± 48.6
7	oral	2591.3 ± 875.5	26.9 ± 27.4	3383.8 ± 1818.8	67.8 ± 58.5	7267.5 ± 4006.6	76.2 ± 32.2
8	SC	NS	98.2 ± 60.7	NS	233.6 ± 215.8	NS	723.3 ± 250.8
10	SC	1124.1 ± 325.9	79.1 ± 51.5	1481.3 ± 359.7	263.1 ± 249.4	2461.3 ± 1075.8	390.2 ± 236.2
11	oral	NS	34.9 ± 36.2	NS	66.6 ± 57.4	NS	99.2 ± 36.3
14	oral	NS	19.0 ± 7.3	NS	53.8 ± 59.0	NS	79.1 ± 38.0
17	oral	2811.3 ± 1502.1	42.7 ± 49.7	4160.0 ± 1166.0	64.5 ± 54.7	5798.8 ± 3218.0	108.5 ± 67.8
28	SC	NS	117.2 ± 86.1	NS	236.6 ± 130.2	NS	394.8 ± 187.7
30	SC	1358.6 ± 254.5	94.8 ± 68.3	1821.6 ± 817.9	326.0 ± 276.7	2878.8 ± 831.4	418.7 ± 246.2
31	oral	NS	27.2 ± 16.0	NS	101.5 ± 152.6	NS	94.7 ± 69.7
34	oral	NS	35.3 ± 28.5	NS	92.2 ± 58.5	NS	84.2 ± 30.3
37	oral	2717.6 ± 1589.6	90.4 ± 106.6	4009.4 ± 1765.2	80.0 ± 62.6	6460.0 ± 3839.3	123.2 ± 121.7

*SD* standard deviation, *SC* subcutaneous, *NS *not sampled

### Pathology

All animals were normal upon macroscopic observation with the exception of cysts involving the uterus in a 3X female and oviducts in a 2X female. There were no definitive robenacoxib-related organ weight changes. In the pooled statistical evaluations, only the liver showed statistically significant changes, where absolute liver weight was increased by 14% in the 3X group (*p* = 0.0174), and the ratio of liver weight to body and brain weight was increased in the 2 × (11% [*p* = 0.0104], 14% [*p* = 0.0385], respectively) and 3X groups (16% [*p* = 0.0006], 19% [*p* = 0.0072], respectively) compared to control. There were no microscopic correlates for the liver weight changes. In the non-pooled statistical evaluations of organ weight changes, only the absolute lung weights and lung/brain weight ratio showed statistically significant changes. These changes were limited to the 1X and 2X dose groups, with the direction of the change opposite between sexes (decreased in males and increased in females). There were no microscopic correlates for the lung weight changes.

All fluctuations among bone marrow components were considered within an acceptable range for biologic variability including one female in the 1X group and one female in the 2X group that had slight fluctuations among myeloid/erythroid ratio and lymphoid cells. Robenacoxib-related microscopic findings were limited to increased injection site subacute/chronic inflammation in all treated groups. The injection site subacute/chronic inflammation was limited to the subcutaneous adipose tissue and was characterized by lymphocytes, monocytes, macrophages and small numbers of neutrophils as well as deposition of fibrous connective tissue. Occasionally the inflammatory reaction contained a small central cystic region. There were no microscopic abnormalities in the overlying skin (dermis, hair follicles, adnexa, epidermis) or underlying muscle. All of the injection site inflammation observations at 1X dose were minimal in severity and generally only slightly increased in incidence compared to the incidence in the control group. The injection site observations from sites injected on Days 8 to 10 typically had a less severe inflammatory reaction compared to the injection sites injected on Days 18 to 20 and 28–30, which was considered to be due to the greater recovery interval for these sites prior to necropsy collection on Day 38. The low incidence and severity of the inflammation observed at sites injected on Days 8–10 in 1X animals was considered to be indicative of almost complete resolution by the Day 38 necropsy. All other microscopic observations, including a tongue ulceration in a 1X female, were considered incidental in nature as they were not associated with any organ weight changes, clinical signs, clinical pathology, or definitive dose relationship; and can sometimes be observed as background (spontaneous) findings.

## Discussion

Individually, the safety of the oral and injectable commercial formulations of robenacoxib in cats have been demonstrated in support of their regulatory approvals [12–14]. However, the safety of switching between the two formulations had to be demonstrated to allow for interchangeable use. The objective of this study was to evaluate the safety of the interchangeable use of robenacoxib in cats when administered orally and subcutaneously at the clinical dosages (1X) as well as at exaggerated doses of two (2X), and three (3X) times the upper limit of the clinical oral dose band (2.4 mg/kg) and the subcutaneous daily dose (2 mg/kg). The dose levels and dose multiples (up to 3X) were chosen in accordance with Target Animal Safety Guidelines for Veterinary Pharmaceutical Products (VICH GL43 [15]) to identify a margin of safety. This modification from the traditional approach of using 1X, 3X and 5X dose levels in target animal safety studies was based on consideration of animal welfare and avoided generation of data already available. A similarly designed study has been performed in dogs [16]. A further objective of the study was to demonstrate the safety of robenacoxib in young (4 months old) cats.

Alternating regimens of robenacoxib tablets (for 7 days) and injection (for 3 days) for three cycles followed by an additional 7 days of oral treatment, providing a total treatment duration of 37 days was well-tolerated. The cats remained in overall good health with treatment-related abnormalities confined to injection site changes and prolongation of the QT interval. As these effects have been seen previously [12–14] with administration of the oral tablets or subcutaneous injection individually, neither should be attributed to their interchangeable use.

The principal targets for toxicity of NSAIDs are the gastrointestinal tract, kidney, liver and inhibition of blood clotting [17, 18]. There was no evidence of robenacoxib toxicity on the liver or kidney. Despite a small increase in liver weight, there were no macroscopic changes nor histologic correlates. There were no effects on alkaline phosphatase, alanine and aspartate aminotransferases, GGT, creatinine, and BUN compared to baseline or to controls. Gastrointestinal findings in this study were limited to observations of vomiting or soft stools which were either observed sporadically across groups or not correlated to changes in body weight or microscopic changes. One cat in the 1X group developed an oral ulcer that was unlikely to be treatment related as it was observed in a single animal and no dose dependency was apparent. Oral ulceration has been reported in humans treated with the sulphone coxib rofecoxib [19] so the possibility that this observation could be a result of exaggerated pharmacology or a treatment effect cannot be completely ruled out. There was no effect of robenacoxib treatment on coagulation parameters prothrombin time, activated partial thromboplastin time and fibrinogen. One 2X cat had an approximately seven-fold increase in the BMBT value compared to the pre-treatment value. The relationship to treatment with robenacoxib could not be established since the prolonged BMBT was only observed in one animal and there was no dose response or correlation to hematology or coagulation parameters at Day 37 in this animal.

Injections were rotated among nine different sites to prevent multiple injections at the same site (as recommended by the Joint Working Group on Refinement [20]). Despite this, robenacoxib-related injection site changes were observed but were minimal or mild and typically had resolved by 72 hours. Macroscopic observations were limited to edema and were more common in 2X and 3X animals. These observations are consistent with a previous target animal safety study that evaluated robenacoxib solution for injection [13] in healthy adult domestic short-hair cats at 0 (control) and 10 mg/kg of robenacoxib for three consecutive days. There were transient, fluid-filled, painless enlargements at the injection sites that disappeared over a period of 72 hours. Microscopic findings consisting of a minimal subcutaneous inflammatory cell infiltration were considered related to robenacoxib. Elevated creatine kinase was observed in the three day target animal safety study as well as in the current study. This finding was considered typical of procedure-related handling but also may have been related to the injection site reactions observed. The level of elevated creatine kinase was not considered related to robenacoxib based on the small magnitude of the changes and overlap with values observed in controls.

The QT interval is influenced by heart rate, thus the reason the correction formulae for QT duration (QTc) in human cardiology take into account RR interval. Unfortunately, no validated QT correction formula exists for the cat. The relationship between heart rate and QT was evaluated in twenty cats that were Holter-monitored for 24 h [21]. A quadratic relationship was discovered with the resultant formula: QT interval duration = 0.41845798 - (0.00181963 X HR) + 0.00000313 X HR^2^). In this study, HR ranged from 90 to 275 bpm. At heart rates above this range (as was seen in the current study) the predicted QT interval increases with increasing heart rate, therefore the equation is no longer applicable. Normal heart rate in the cat has been referenced as 140–220 bpm in adult cats and 220–260 bpm in kittens. In the current study, cats were approximately 4 months of age yet had heart rates well above what would be considered physiologic. The sedation protocol may not have been optimal for ECG recording as the onset of action of ketamine occurred earlier than that of acepromazine. Ketamine alone can cause psychomotor effects with muscle hypertonia, agitation, possible hallucination, and a high sympathetic stimulation increasing heart rate and sinus rhythm. Acepromazine does not cause muscle relaxation to counteract such increased motor activity. One must therefore be cautious making conclusions regarding the ECG in general and QT specifically.

To account for the effect of heart rate on QT duration, the terminal RR interval was used as a covariate. By doing so, a dose-dependent increase in QT duration was observed at the exaggerated dose levels with an increase relative to the control of 6.3% and 7.5% in the 2X and 3X groups, respectively. No prolongation was observed at the 1X dose. The clinical relevance of such QT interval prolongation in cats is unknown as studies of drugs known to cause QT prolongation with risk of Torsade de Pointe (TdP) have not been extensively performed in cats. A group of five antipsychotic drugs were shown to cause dose-dependent QT prolongation, in some cases by more than 50%, in the perfused isolated feline heart model [22]. Because these compounds are not used clinically in cats, it is impossible to know if they would cause TdP or other cardiac arrhythmias. The class III antiarrhythmic agent, almokalant, was tested for its ability to prolong the QT interval in anesthetized rabbits and cats [23]. At the same infusion rate, TdP was initiated in nine of 10 rabbits, whereas it was only seen in one of six cats, suggesting cats may be less sensitive. Almokalant caused a statistically significant lengthening of the QT interval in cats from 241 ± 6.0 ms to a maximum of 349 ± 8.0 ms (i.e., by approximately 45%). These results prompted the authors to conclude that no obvious relationship exists between any critical QT lengthening and proarrhythmias. The magnitude of the effects seen in the current study were well below that seen with almokalant, thus it seems unlikely that there is a clinically significant risk of cardiac arrhythmmogenicity of robenacoxib.

There have been no direct evaluations of robenacoxib’s ability to increase the QT interval, therefore indirect evidence must be obtained from the various studies (e.g., safety and efficacy) performed during its development. When a single dose of robenacoxib was administered SC at 2 mg/kg or at 2 or 4 mg/kg IV to anesthetized cats, no electrocardiographic changes (including QT interval) were detected at 5 or 60 min post administration [24]. Although dose-dependent QT prolongation was seen in the six-month target animal safety study of oral robenacoxib administered at 1, 3 and 5X, no arrhythmias were documented [12]. Additionally, adverse events indicative of cardiac arrhythmia or TdP were not reported in cats in any other safety study, clinical trial [10, 25], nor during post-marketing pharmacovigilance.

Interpretation of the toxicokinetic results in this study is made difficult by two study design factors, the limited sampling schedule and the dosing regimen. A maximum of three (one predose and two post dose) samples during a dosing interval is insufficient to accurately estimate T_max_, C_max_ half-life, or AUC. Although accurate dosing was achievable with subcutaneous injection, because of the minimum dose increment available (i.e., 6 mg whole tablet), a broad oral dose range occurred where the highest individual dose received in a lower dose group was almost identical to the lowest individual dose received in the higher dose group (e.g., 4.7 mg/kg in the 1X group vs. 4.8 mg/kg in the 2X group).

Lower 0.75 h concentrations were observed following subcutaneous compared to oral administration, however, injection site inflammation as well as the large dose volumes used for establishing safety in the current study may have impacted rate and/or absorption of robenacoxib subcutaneously.

No robenacoxib accumulation was observed in this 37-day study. Previous studies have shown robenacoxib to be eliminated in the cat with a half-life of less than 2 hours [26, 27], therefore no accumulation would be expected with once daily dosing. Ultimately, the toxicokinetic analysis confirmed the relevant exposure of the animals in each dose group and thus allows appropriate conclusions about the safety of the interchangeable use of the two formulations.

It should be emphasized that the study reported in this paper, as with all target animal safety studies required for product registration, was conducted in healthy young cats. Because adverse effects of NSAIDs can be exacerbated by the physiological state of the animal, such as hydration status, or in older cats or cats with pre-existing damage to the gastrointestinal tract or kidney, the same results might not have been observed. The safety of NSAIDs needs to be evaluated in both target animal safety and field studies. Indeed robenacoxib was found to be well tolerated in cats undergoing surgery and with osteoarthritis [10, 11, 25, 28–30].

## Conclusions

The safety and efficacy of two different approved formulations of robenacoxib, given by two different routes and regimens, have been demonstrated individually. However, there are clinical situations in which administration of both formulations might be indicated. It was therefore necessary to demonstrate their safe interchangeable use in this study by administering several multiple daily dose cycles of each formulation at exaggerated doses. The study found no treatment-related changes in body weight, food consumption, ophthalmic or physical examinations, BMBT, clinical pathology or organ weight. Findings attributed to robenacoxib treatment were confined to injection site changes (transient edema with minimal or mild, subacute/chronic inflammation histologically), and prolongation of the QT interval on ECG evaluation and neither had a clinically significant effect on the health or well-being of the animals. Both of these findings have been observed with the individual use of robenacoxib tablets or solution for injection or are expected with this class of compound and mode of administration, and therefore are not associated with the interchangeable use of these two products. This study demonstrates that interchangeable use of robenacoxib injection and tablets is safe in cats when used for approved indications according to the labeled directions for each product. The ability to initiate therapy with one form and maintain it with the other provides the flexibility that veterinarians and owners require.

## Methods

### Objective and standards

The objective of the study was to establish the safety of the interchangeable use of robenacoxib tablets and solution for injection in cats.

This study was designed in consultation with US regulatory authorities (FDA-CVM) and according to VICH Guideline GL 43 “Target Animal Safety—Pharmaceuticals: Target Animal Safety for Veterinary Pharmaceutical Products” [15]. The study was conducted in compliance with Good Laboratory Practice quality standards [31, 32], with the Animal Welfare Act [33], the Guide for the Care and Use of Laboratory Animals [34], and the Office of Laboratory Animal Welfare [35]. All animal procedures were reviewed and approved by the study site Institutional Animal Care and Use Committee (Protocol 382 − 208).

### Animal management

Thirty-four (17 male and 17 female) domestic short-hair cats obtained from a commercial source (Liberty Research, Inc., Waverly, New York, USA) were used. The number of animals was determined based on recommendations set forth in VICH GL 43. Each animal was uniquely identified with a microchip and via tattoo. The cats weighed 1.55 to 2.75 kg seven days prior to dosing and were approximately 4-months old (121 to 125 days) on Day 1, the first day of dosing. Animals were in good health at the start of the study based on veterinary examination and clinical pathology screening during the 20-day acclimation period. Cats were singly housed in stainless steel cages with plastic coated flooring equipped with a litter box and resting perch in a building environmentally controlled to provide approximately 12 hours of light, temperature of 18 to 29 °C (64 to 84°F) and humidity of 30 to 70%. Cats were given *ad libitum* access to tap water and environmental enrichment in the form of various cat toys. A commercial dry cat food (Laboratory Feline Diet #5003, PMI Nutrition International, Inc.) was fed to the animals in a ration calculated to maintain a healthy body weight. Beginning on Day − 1, and for the duration of the study, the animals were fasted overnight for at least 8 hours, but not greater than 12 hours, and offered food 2 to 3 hours following dose administration.

### Experimental design

A randomized controlled study design was used and all personnel involved in collecting live phase animal data were blinded to treatment group allocation. Animals were randomized to treatment groups (Table 6) and to cages using a simple randomization procedure within each sex. The study lasted 37 days in total and consisted of alternating sequences of daily robenacoxib dosing using tablets (for 7 days) or subcutaneous (SC) injection (for 3 days) as outlined in Table 7. Groups were treated with a control and 1X, 2X, and 3X the maximum recommended dose of robenacoxib tablets or injection as described in Table 6. The maximum recommended 1X dose is the highest dose that an animal can receive based on the approved label (i.e., 6 mg for a 2.5 kg cat).

**Table 6:** Experimental groups used to evaluate the safety of interchangeable use of oral and subcutaneous administration of robenacoxib in cats

**Group**	**Treatment**	**Multiples of MRD**^**a**^	**Target daily oral dose level (mg/kg)**	**Target daily subcutaneousdose level / dose volume(mg/kg) / (mL/kg)**	**Number of cats**	
					**Males**	**Females**
1	Control^b^	0	0	0 / 0.3	5	5
2	Robenacoxib	1X	≥ 2.4	2.0 / 0.1	4	4
3	Robenacoxib	2X	≥ 4.8	4.0 / 0.2	4	4
4	Robenacoxib	3X	≥ 7.2	6.0 / 0.3	4	4

^a^*MRD* maximum recommended dose

^b^Two empty capsules orally, sterile saline subcutaneously

**Table 7:** Study design used to evaluate the safety of interchangeable use of oral and subcutaneous administration of robenacoxib in cats

**Dosing Cycle**	**Study Day**	**Route of administration**	**Dosing Regimen x (Multiplication Factor**^**a**^)
1	1 to 7	Oral	≥2.4 mg/kg/day X (0,1,2,3)
	8 to 10	SC	2.0 mg/kg/day X (0,1,2,3)
2	11 to 17	Oral	≥2.4 mg/kg/day X (0,1,2,3)
	18 to 20	SC	2.0 mg/kg/day X (0,1,2,3)
3	21 to 27	Oral	≥2.4 mg/kg/day X (0,1,2,3)
	28 to 30	SC	2.0 mg/kg/day X (0,1,2,3)
4	31 to 37	Oral	≥2.4 mg/kg/day X (0,1,2,3)

^a^Multiplication factors were applied to the dose in treated groups (i.e. Group 4 corresponds to 3X the MRD or 3 X ≥2.4 mg/kg/day = ≥7.2 mg/kg/day oral administration)

### Test articles and administration

Treated animals received Onsior (robenacoxib) 6 mg Tablets for Cats inside gelatin capsules orally (to facilitate administration) and Onsior (robenacoxib) injection (20 mg/ml injection For Subcutaneous Use In Cats) subcutaneously. Both formulations were the final commercial products manufactured according to Good Manufacturing Practices. The number of oral tablets was calculated to ensure that all animals received at least the targeted dose level for that group (Table 6). For example, for Group 2, this meant that cats received an oral robenacoxib dose of at least 2.4 mg/kg. Robenacoxib solution for injection was delivered at the exact recommended dose level (i.e., 2 mg/kg for Group 2). Control animals received two empty gelatin capsules on oral dosing days and 0.3 mL/kg sterile saline on SC dosing days. The injection sites were rotated among the left shoulder (Days 8, 18, and 28), the midline intrascapular region (Days 9, 19, and 29), and right shoulder (Days 10, 20, and 30). Additionally, three different locations oriented cranially to caudally within each region were used. Dose administration occurred each day at approximately the same time (i.e., early morning).

### Experimental measures

#### Cageside and detailed clinical observations, body weight, food consumption, injection site monitoring

Cageside observations were performed twice daily on all animals for morbidity, mortality, injury, and the availability of food and water. Detailed clinical observations were performed by trained technical staff twice per day beginning on Day − 2. Observations were at least six hours apart with the first occurring 1–2 hours after dose administration. Observations included, but were not limited to eyes, mucus membranes, respiratory system, circulatory system, autonomic and central nervous systems, somatomotor activity and behavior pattern. Body weight was measured on Day − 19 and weekly beginning Day − 14. Individual food consumption was measured daily beginning on Day − 7. Injection sites were evaluated for erythema, edema, heat, and pain response (i.e., vocalization upon palpation using a 3-point Likert scale) on each day of subcutaneous dosing prior to and 7 to 8 hours after dosing and once daily for three days following each dose administration. If any injection site was abnormal at the end of the observation period, additional assessments were carried out until the cat returned to normal or until end of the study.

#### Ophthalmoscopic, physical, neurological, and electrocardiographic examinations

Ophthalmoscopic examinations were conducted on all animals on Day − 3 and Day 33 by a board-certified veterinary ophthalmologist. A complete physical examination with neurological assessment was conducted on all animals by a licensed veterinarian on Days − 14, -6, 10 and 36. The assessments of toxicity and health included general condition and behavior, general ocular without ophthalmoscope, integument, musculoskeletal, gastrointestinal, body temperature, cardiovascular and respiratory including assessment by auscultation, and reproductive system. The neurological assessment included observation of nystagmus, pupillary response, extensor thrust (muscle tone), righting reflex, startle reflex, and walking movement. Particular attention was given to assessments of the central nervous system for signs of seizures, tremors, salivation, vomiting, depression, lethargy, ataxia, mydriasis, and diarrhea. All animals received an electrocardiographic examination under sedation with ketamine (approximately 13–19 mg/kg, intramuscularly) and acepromazine (approximately 0.2–0.6 mg/kg, subcutaneously) on Days − 5 and 36. Standard ECGs (10 Leads) were recorded at 50 mm/sec. The ECG was assessed qualitatively as well as quantitatively by a board-certified veterinary cardiologist. Using an appropriate lead, the RR, PR, and QT intervals, and QRS duration were measured and heart rate was calculated. Heart rate was calculated from the average of five RR intervals. The QT interval was not was corrected to changes in heart rate because this procedure has not been validated for cats.

#### Buccal mucosal bleeding time, hematology, coagulation, clinical chemistry, urinalysis

Buccal mucosal bleeding times (BMBT) were measured on all animals on Days − 5 and 36 following ECG examinations while the animals were sedated. The upper lip was everted and a Surgicutt incision device was used to create a small incision (approximately 1 mm in depth and 3.5 mm in length). Blood flow from the incision site was blotted using filter paper below the incision. The elapsed time from the triggering of the device until blood no longer appeared on the filter paper was recorded as the bleed time. Clinical pathology evaluations were conducted on all animals on Days − 13 and − 6 and prior to dosing on Days 13 and 37. Because reference values given by the laboratory were (slightly) different for males and females, they were combined (i.e. lower of the two lower bounds to higher of the two upper bounds) for the tables. Animals were fasted 8–12 hours overnight prior to blood collection. Blood samples (3.0 mL) were collected from the jugular vein into tubes containing K_3_EDTA for evaluation of hematology parameters, sodium citrate for evaluation of coagulation parameters, and serum separators with no anticoagulant for the clinical chemistry samples. Urine samples were collected on Days 13, 6, 13, and 37 by replacing the litter in the litter box with NoSorb® (Creative Science, LLC, Ballwin, MO, USA) for at least 16 hours. Hematology variables were determined using an Advia 120 or Advia 2120 (Siemens, Munich, Germany), light microscope, or sediplast column (Polymedco, NY, US). Coagulation variables were determined using a Stago Compact (Diagnostica Stago, Parsippany, NJ, US), clinical chemistry variables were determined using an Olympus AU2700 or AU640e (Olympus Diagnostics, Southall, UK), and urinalysis variables were determined using a Clinitek-500 or Atlas (Siemens, Munich, Germany), Reichert VET 360 (Reichert Technologies, Buffalo, New York, USA), or light microscope.

#### Analyses of blood samples for robenacoxib concentration

Approximately 0.5 mL of whole blood was collected from animals via the jugular vein and placed in tubes containing K_2_EDTA. Samples were collected within 30 minutes prior to dosing and at 0.75, and 4 hours postdose on Days 1, 4, 7, 10, 17, 30, and 37. A single 0.5 mL sample was collected from all animals 4 hours postdose on Days 8, 11, 14, 28, 31, and 34. The animals were fasted for predose and 0.75 hour blood collections and were fed 2 to 3 hours after dose administration. Samples were stored frozen (-10 to 30 °C) within approximately 1 hour after collection until analyzed. Day 1 blood samples were inadvertently processed to plasma and therefore not analyzed.

Robenacoxib was quantitatively analyzed in blood using a validated analytical method involving liquid chromatography with tandem mass spectrometry detection (LC-MS/MS) that has been previously describe [16]. Performance of the method was monitored by low, medium and high quality control samples, where inter-run %RSD (relative standard deviation) was 8.3, 7.4, and 5.8, respectively.

### Toxicokinetics of robenacoxib

Only abbreviated toxicokinetic sampling was performed so as not to interfere with the primary objective of the study. Concentrations below the limit of quantification were considered 0. The limited sampling schedule did not allow appropriate estimation of standard PK parameters and thus mean concentration data were calculated and compared by route, timepoint and dose group.

### Pathology

Necropsies were performed in cage order. All animals were euthanized on Day 38 by intramuscular sedation with ketamine followed approximately 30 min later in a different room by an intravenous overdose of sodium pentobarbital solution and exsanguination, under procedures approved by a veterinary pathologist and according to the study site’s standard operating procedures. After a complete post-mortem evaluation, tissue samples were collected for histopathological analysis. Additionally, the following organs were weighed before tissues were collected: adrenal gland, aorta, brain, epididymis, heart, kidney, liver, lung with bronchi, ovary, pituitary, prostate, salivary gland (mandibular), spleen, testis, thymus, thyroid gland (with parathyroid), and uterus with cervix. In order to correct for body weight and size, the ratio of organ weight to body weight and brain weight was calculated for each animal. Tissues, including any gross lesions identified during the necropsy, were fixed in neutral buffered formalin, except the eye including optic nerve and testes were fixed using a modified Davidson’s fixative [36]. Tissue sections were stained with hematoxylin and eosin and evaluated by a board-certified veterinary pathologist. A bone marrow smear was collected from a rib at necropsy and analyzed qualitatively by a board-certified veterinary clinical pathologist.

### Statistical analysis

The experimental unit used was the individual cat.

#### Hematology, coagulation, clinical chemistry, body weights, food consumption

Post-treatment values were analyzed using RMANCOVA, with treatment group, sex, time, and their interactions as fixed model effects and the pre-treatment value measured closest to dosing as a covariate. Repeated measurements in the same animal were modeled using a compound symmetry or heterogeneous compound symmetry correlation structure (for food consumption: autoregressive first-order or heterogeneous autoregressive first-order); the model with the smallest Akaike’s Information Criterion was selected for each outcome.

If any of the interactions involving treatment was significant (p < 0.1 for the two-factor interactions or p < 0.05 for the three-factor interaction), it was inappropriate to evaluate the main or overall treatment effect independently of the interacting factor and, as such, treatment comparisons were conducted for each level of the interacting factor. If none of the interactions involving treatment were significant, overall treatment differences were assessed. If a significant treatment effect was observed, pairwise differences between the 1X, 2X and 3X groups and the placebo group were assessed, without adjustment for multiple comparisons.

#### ECG

Heart rate and QT interval were analyzed using ANCOVA, with treatment group, sex, and their interaction as fixed model effects. For HR, pre-treatment HR was used as a covariate. However, because the QT interval is influenced by HR (RR interval), pre-treatment QT is not appropriate as a covariate, and therefore only the terminal RR value was used as the covariate in the analysis of QT interval.

If the treatment x sex interaction was significant (p < 0.1), treatment comparisons were conducted separately for males and females. If the interaction was not significant, overall treatment differences were assessed using pooled data from males and females. Significant treatment effects were further evaluated using unadjusted pairwise comparisons between the 1X, 2X and 3X groups and the placebo group.

#### Organ weights

Values were analyzed using ANOVA, with treatment group, sex, and their interaction as fixed model effects and the pre-treatment value as a covariate.

If the treatment x sex interaction was significant (p < 0.1), treatment comparisons were conducted separately for males and females. If the interaction was not significant, overall treatment differences were assessed, pooling data from males and females. Significant treatment effects were further evaluated via unadjusted pairwise comparisons between the 1X, 2X and 3X groups and the placebo group.

## Data Availability

All data cannot be freely shared without control due to confidentiality. Please contact the corresponding author to request access to non-confidential data.

## Abbreviations

ANOVA: Analysis of variance
ANCOVA: Analysis of covariance
AUC: Area under the curve
BMBT: Buccal mucosal bleeding time
bpm: Beats per minute
BUN: Blood urea nitrogen
COX: Cyclooxygenase
C_max_: Maximum blood drug concentration
ECG: Electrocardiogram
EMA CVMP: European Medicines Agency Committee for Medicinal Products for Veterinary Use
EU: European Union
FDA-CVM: Food and Drug Administration’s Center for Veterinary Medicine
GGT: Gamma-glutamyl transferase
HPLC: High-performance liquid chromatography
K_2_EDTA: Dipotassium ethylenediaminetetraacetic acid
K_3_EDTA: Tripotassium ethylenediaminetetraacetic acid
LC-MS/MS: Liquid chromatography tandem mass spectrometry
MRD: Maximum recommended dose
ms: Milliseconds
NSAID: Non-steroidal anti-inflammatory drug
RMANCOVA: Repeated measures analysis of covariance
RSD: Relative standard deviation
SC: Subcutaneous
T_max_: Time to maximum blood concentration following drug administration
TdP: Torsades de pointes
US: United States of America
VICH: Veterinary International Conference on Harmonization
